# Dynamic Control of *ERG20* and *ERG9* Expression for Improved Casbene Production in *Saccharomyces cerevisiae*

**DOI:** 10.3389/fbioe.2018.00160

**Published:** 2018-11-01

**Authors:** Roberta Callari, Yvan Meier, Davide Ravasio, Harald Heider

**Affiliations:** Evolva SA, Reinach, Switzerland

**Keywords:** casbene, diterpene, *ERG20*, *ERG9*, yeast, metabolic engineering, dynamic control, mevalonate pathway

## Abstract

Production of plant metabolites in microbial hosts represents a promising alternative to traditional chemical-based methods. Diterpenoids are compounds with interesting applications as pharmaceuticals, fragrances and biomaterials. Casbene, in particular, serves as a precursor to many complex diterpenoids found in plants from the Euphorbiaceae family that have shown potential therapeutic effects. Here, we engineered the budding yeast *Saccharomyces cerevisiae* for improved biosynthesis of the diterpene casbene. We first expressed, in yeast, a geranylgeranyl diphosphate synthase from *Phomopsys amygdali* in order to boost the geranylgeranyl diphosphate pool inside the cells. The enzyme uses isopentenyl diphosphate and dimethylallyl diphosphate to directly generate geranylgeranyl diphosphate. When co-expressing a casbene synthase from *Ricinus communis* the yeast was able to produce casbene in the order of 30 mg/L. Redirecting the flux from FPP and sterols, by means of the ergosterol sensitive promoter of *ERG1*, allowed for plasmid-based *casbene* production of 81.4 mg/L. Integration of the target genes into the yeast genome, together with the replacement of the promoter regions of *ERG20* and *ERG9* with combinations of ergosterol- and glucose-sensitive promoters, generated a titer of 108.5 mg/L of casbene. We here succeeded to engineer an improved route for geranylgeranyl diphosphate synthesis in yeast. Furthermore, we showed that the concurrent dynamic control of *ERG20* and *ERG9* expression, using ergosterol and carbon source regulation mechanisms, could substantially improve diterpene titer. Our approach will pave the way for a more sustainable production of GGPP- and casbene-derived products.

## Introduction

Diterpenoids represent one of the largest and most diverse classes of plant metabolites. Although in some cases they carry out important primary functions (e.g., regulation of growth and development by gibberellins), they are usually products of the secondary metabolism with specialized pathways extremely varied across the plant kingdom (Zerbe and Bohlmann, 2015). Diterpenoids are beneficial for plants because of their role in protection from abiotic stress and control of the ecological interactions with other organisms (e.g., defense against herbivores and microbial pathogens) (Cheng et al., 2007; Tholl, 2015). Moreover, they can benefit humanity because of their industrial application as pharmaceuticals (e.g., paclitaxel, forskolin, ingenol-3-angelate, prostratin), fragrances (e.g., sclareol), and other industrial bioproducts (e.g., steviol glycosides as natural sweetners, diterpene resins for inks, and coatings) (Bohlmann and Keeling, 2008; Goyal et al., 2010; Caniard et al., 2012; Doseyici et al., 2014; Fidler and Goldberg, 2014; Howat et al., 2014; Miana et al., 2015).

Current production methods of diterpenoids rely on extraction from natural sources and chemical synthesis. Because of the low yield occurrence in the producing organisms and the structural complexity of the compounds, such methods are inefficient and environmentally costly. An attractive and environmentally friendly alternative is represented by microbial fermentation. Biosynthesis in heterologous hosts such as *Escherichia coli* or *Saccharomyces cerevisiae* can (1) reduce costs using sugar-based carbon sources, (2) increase sustainability by avoiding harvesting and extraction from natural sources, (3) increase yield and productivity using genetic manipulation of the heterologous hosts, and (4) provide enantiomerically pure products through enzymatic biocatalysis (Scalcinati et al., 2012). *S. cerevisiae* in particular is a robust host that not only offers the biosynthetic machinery needed for production of diterpenoids, but also contributes the necessary environment for expression of membrane-bound enzymes, such as cytochrome P450 hydroxylases. These p450 enzymes are frequently involved in the biosynthesis of complex plant terpenoids and are usually difficult to express in prokaryotic systems (Hamann and Møller, 2007; Kirby and Keasling, 2009).

**Figure 1:**
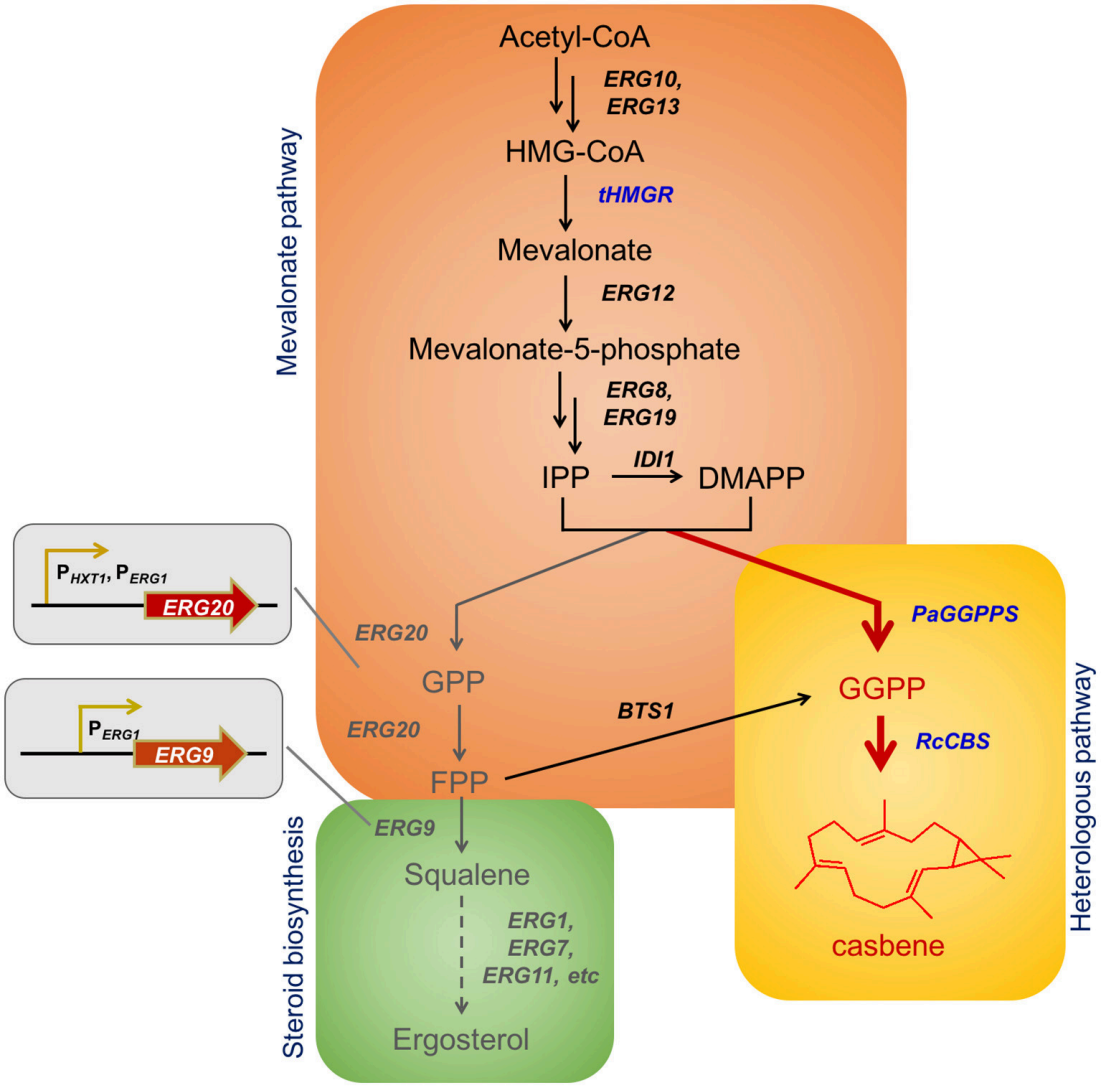
Biosynthesis of casbene in yeast and examples of casbene derived compounds. Schematic overview of casbene biosynthesis based on engineered mevalonate pathway in *S. cerevisiae*. The new biosynthetic branch starts with *PaGGPPS*, for production of GGPP using IPP and DMAPP as sole substrates. *RcCBS* mediates casbene synthesis (in red). Endogenous genes and metabolites are shown in black. Overexpressed genes are shown in blue. Genes regulated through dynamic control (*ERG20* and *ERG9*) and genes from the steroid biosynthetic pathway are shown in gray. The truncated endogenous gene *tHMG1* and the heterologous genes *PaGGPPS* (encoding a truncated version of the fusicoccadiene synthase from *Phomopsis amygdali* serving as GGPP synthase) and *RcCBS* (encoding a truncated version of the casbene synthase from *Ricinus communis*) were overexpressed to improve casbene production. The native promoter of *ERG20* was replaced with P_*ERG*1_ or P_*HXT*1_, respectively. The native promoter of *ERG9* was replaced with P_*ERG*1_. HMG-CoA, 3-hydroxy-3-methylglutaryl coenzyme A; IPP, isopentenyl pyrophosphate; DMAPP, dimethylallyl pyrophosphate; GPP, geranyl diphosphate; FPP, farnesyl diphosphate; GGPP, geranylgeranyl diphosphate.

The precursors for production of terpenes are present in the native metabolic network of *S. cerevisiae* (Chambon et al., 1991). The yeast mevalonate (MVA) pathway, through multiple rounds of condensation of isopentenyl diphosphate (IPP) and dimethylallyl diphosphate (DMAPP), leads to generation of geranyl diphosphate (GPP), farnesyl diphosphate (FPP) and geranylgeranyl diphosphate (GGPP) (Figure 1). GPP, FPP, and GGPP represent the universal precursor units of all monoterpenes (C_10_), sesquiterpenes (C_15_), and diterpenes (C_20_) respectively. However, the GGPP content in *S. cerevisiae* is rather low, due to the fact that the endogenous geranylgeranyl diphosphate synthase (GGPPS) Bts1p does not compete efficiently with enzymes directly upstream and downstream (farnesyl diphosphate synthase Erg20p and squalene synthase Erg9p) for the pools of IPP and FPP (Jiang et al., 1995). Thus, the endogenous flux of the pathway favors production of FPP, and the sterols derived from it. As a consequence, improvement of the GGPP pool is essential for efficient, high-titer diterpene production. To reach this goal different studies have focused on either the use of heterologous GGPP synthases, Erg20p mutants that are able to synthetize GGPP, or on fusions of *BTS1* and *ERG20* (Kirby et al., 2010; Zhou et al., 2012; Ignea et al., 2015; Song et al., 2017). Such strategies have been shown to improve production of GGPP-derived compounds, but they have also demonstrated that additional improvements in substrate conversion toward diterpene production are feasible.

**Figure 2:**
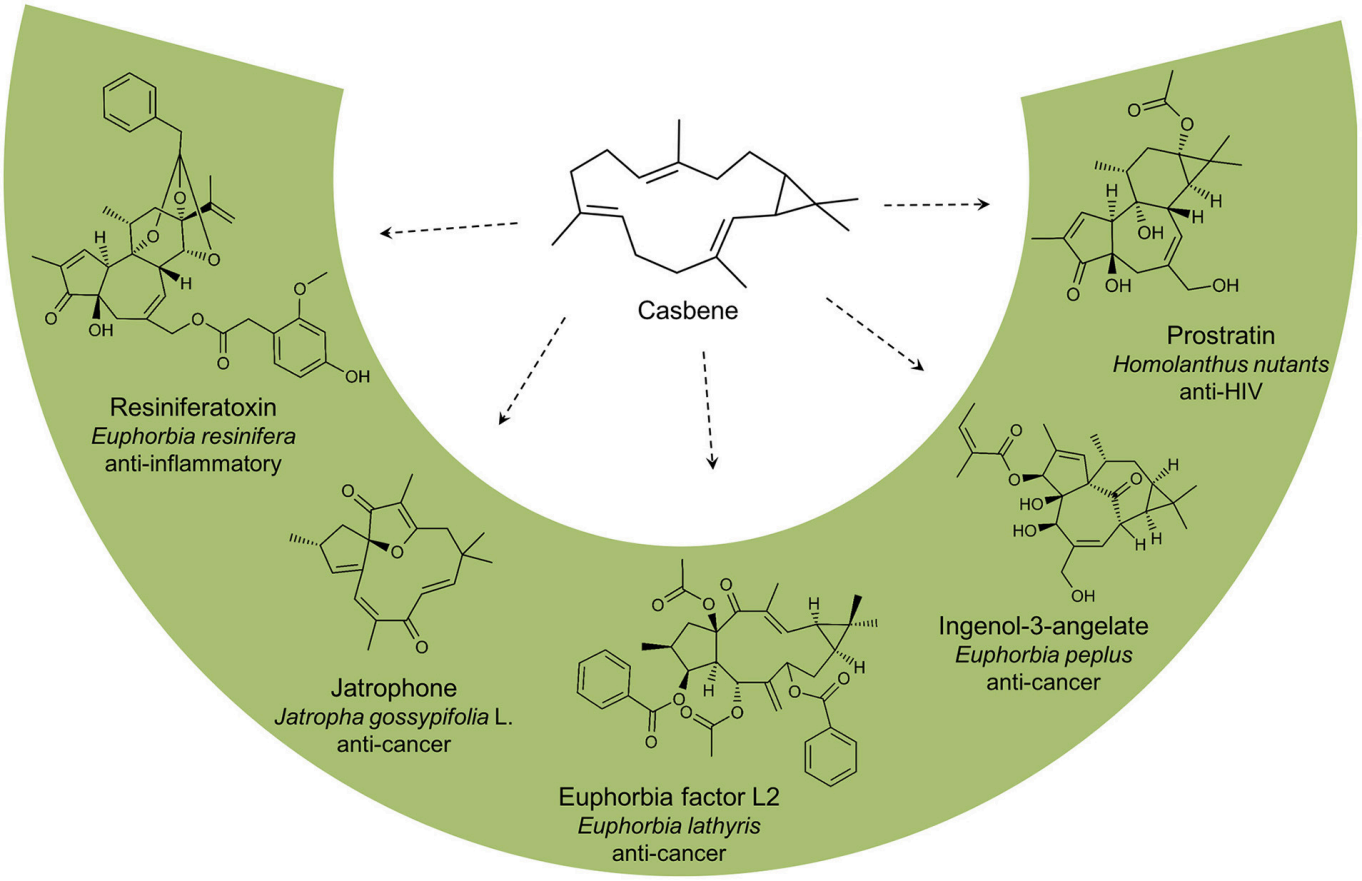
Casbene is the precursor of many diterpenoids identified in plants from the Euphorbiaceae family. Euphorbia factor L2 (*Euphorbia lathyris*), resiniferatoxin (*Euphorbia resinifera*), ingenol-3-angelate (*Euphorbia peplus*), prostratin (*Homolanthus nutans*), and jatrophone (*Jatropha gossypifolia* L.).

In this study, we developed a yeast platform for production of the diterpene hydrocarbon casbene, originally identified from castor bean (*Ricinus communis*) where it serves as a phytoalexin (Mau and West, 1994). In the last decades, the compound has gained much interest since the conversion of GGPP into casbene, catalyzed by the casbene synthase enzyme, is acknowledged as the first committed step in the biosynthesis of the diterpenoid molecular backbones jatrophane, tigliane, lathyrane, and ingenane (Kirby et al., 2010). Casbene is the precursor of many diterpenoids of medical interest, identified in a number of Euphorbiaceae (Figure 2). For example, Ingenol-3-angelate, found in the sap of *Euphorbia peplus*, has been approved by the FDA for the treatment of actinic keratosis (Fidler and Goldberg, 2014) whilst Prostratin, derived from *Homolanthus nutans* is a protein kinase C activator that inhibits HIV-1 infections and reduces HIV-1 latency (Miana et al., 2015). Jatrophone, isolated from extracts of *Jatropha gossypifolia* L., has shown significant anti-proliferative effects against human tumor cell lines (Kupchan et al., 1970; Theoduloz et al., 2009) and the tigliane derived diterpenoid, resiniferatoxin from *Euphorbia resinifera* is effective against a broad range of inflammatory and neuropathic pain conditions (Iadarola and Gonnella, 2013). Euphorbia factor L2 from *Euphorbia lathyris* induces apoptosis in lung cancer cell lines (Lin et al., 2017).

For efficient production of casbene in yeast, we increased the GGPP supply inside the cells by expression of a truncated, fungal GGPP synthase, from the endophytic fungus *Phomopsis amygdali*. Expression of the enzyme, producing GGPP from IPP and DMAPP directly, significantly improved GGPP biosynthesis and allowed, upon co-expression of the casbene synthase from *R. communis*, for production of casbene in the order of 30 mg/L. Similar titers have been reported previously (Kirby et al., 2010). Dynamic control of genes *ERG20* and *ERG9* by means of ergosterol- and glucose-sensitive promoters further redirected the flux to GGPP and diterpene synthesis, improving casbene production by up to 108.5 mg/L.

We envision that our approach will pave the way toward the sustainable production of various GGPP- and casbene-derived isoprenoids.

## Materials and methods

### Chemicals and media

All chemicals were bought from Sigma-Aldrich (St. Louis Missouri, USA) unless stated otherwise. Authentic standard of cembrene was purchased from CHEMOS GmbH & Co. KG (Regenstauf, Germany). Authentic standard of casbene was received from Professor Birger Møller's laboratory at the University of Copenhagen.

LB medium for growth of *Escherichia coli* was supplied from Carl Roth GmbH + Co. KG (Karlsruhe, Germany), and was supplemented with 100 μg/L of ampicillin for amplification of plasmids. Yeast Extract Peptone Dextrose (YPD) medium with 20 g/L glucose was used for growth of wildtype strains prior to transformation. For preparation of pre- and main cultures we used synthetic complete (SC) drop-out medium (Formedium LTD, Hustanton, England), supplied with 6.7 g/L yeast nitrogen base, 20 g/L glucose and all amino acids necessary for the corresponding auxotrophy.

### Plasmids and strains construction

Table 1 lists all plasmids constructed in this work. Coding sequences (Table S1) were synthesized by GeneArt® (Thermofisher Scientific, Zug, Switzerland) as yeast codon optimized versions. Synthetic genes were cloned via *Hind*III HF, *Sac*II restriction digestion and T4 DNA ligase (New England Biolabs, Ipswich, Massachusetts, USA) according to standard protocols (Green and Sambrook, 2012). *E. coli* XL10 Gold (Agilent, Santa Clara, California, USA) cells were used for subcloning of genes.

**Table 1:** List of plasmids constructed in this work.

**Plasmid**	**Description**	**Plasmid type**
pCAS1	Plasmid harboring expression cassette pTEF1-*RcCBS*-ENO2t	ARS/CEN
pCAS2	Plasmid harboring expression cassette pPGK1-*PaGGPPS*-ADH2t	ARS/CEN
pCAS3	Plasmid for ERG9 promoter exchange with insertion of the promoter region from *ERG1* gene	Integrative
pCAS4	Plasmid for ERG20 promoter exchange with insertion of the promoter region from *CYC1* gene	Integrative
pCAS5	Plasmid for ERG20 promoter exchange with insertion of the promoter region from *ERG1* gene	Integrative
pCAS6	Plasmid for ERG20 promoter exchange with insertion of the promoter region from *HXT1* gene	Integrative
pCAS7	Plasmid for pTEF1-*RcCBS*-tENO2 cassette integration into YOR092W locus	Integrative
pCAS8	Plasmid for pPGK1-*PaGGPPS*-tADH2 cassette integration into YDR122W locus	Integrative

*S. cerevisiae* strains generated throughout this study are listed in Table 2. All constructed strains were derived from strain NCYC 3608 (NCYC, Norwich, United Kingdom), a derivative of strain S288C modified in our labs and carrying a truncated 3-hydroxy-3-methylglutaryl coenzyme A reductase (*tHMGR*) in the YCT1 locus to increase the flow through the early part of the mevalonate pathway (CAS1- MATalpha hoΔ0 his3Δ0 leu2Δ0 ura3Δ0 CAT5(T91M) MIP1(-661T) SAL1 GAL2 YLL055w::loxP-pTDH3-Sc_tHMGR-tCYC1). All yeast strains were stored in 25% glycerol at −80°C. Yeast transformation was performed using the classical lithium acetate method (Gietz and Schiestl, 2007). Transformants were grown on agar plates prepared with selective SC drop-out medium. For integration into the yeast genome, genes were cloned into yeast integration plasmids targeting incorporation into specific sites (Table 1). Transformants were verified by PCR on genomic DNA for correct insertion of heterologous genes.

**Table 2:** List of strains constructed in this work.

**Strains**	**Description**	**MVA/sterols pathway modifications**
H-MEV	S288C derivative with relatively high mevalonate pathway activity	*tHMGR*
CAS1	Strain H-MEV harboring plasmid pCAS1 and empty vector	*tHMGR*
CAS2	Strain H-MEV harboring plasmid pCAS1 and pCAS2	*tHMGR*
CAS3	H-MEV with *ERG20* under the control of P*_*ERG*1_*	*tHMGR*, P*_*ERG*1_*- *ERG20*
CAS4	H-MEV with *ERG9* under the control of P*_*ERG*1_*	*tHMGR*, P*_*ERG*1_*- *ERG9*
CAS5	H-MEV with *ERG20* and *ERG9* under the control of P*_*ERG*1_*	*tHMGR*, P*_*ERG*1_*- *ERG20*, P*_*ERG*1_*- *ERG9*
CAS6	Strain CAS3 harboring plasmid pCAS1 and pCAS2	*tHMGR*, P*_*ERG*1_*- *ERG20*
CAS7	Strain CAS4 harboring plasmid pCAS1 and pCAS2	*tHMGR*, P*_*ERG*1_*- *ERG9*
CAS8	Strain CAS5 harboring plasmid pCAS1 and pCAS2	*tHMGR*, P*_*ERG*1_*- *ERG20*, P*_*ERG*1_*- *ERG9*
CAS9	Strain H-MEV with *ERG20* under the control of P*_*HTX*1_*	*tHMGR*, P*_*HXT*1_*- *ERG20*
CAS10	Strain CAS9 with *ERG9* under the control of P*_*ERG*1_*	*tHMGR*, P*_*HXT*1_*- *ERG20*, P*_*ERG*1_*- *ERG9*
CAS11	Strain H-MEV with integrated *PaGGPPS* and *RcCBS*	*tHMGR*
CAS12	Strain CAS3 with integrated *PaGGPPS* and *RcCBS*	*tHMGR*, P*_*ERG*1_*- *ERG20*
CAS13	Strain CAS9 with integrated *PaGGPPS* and *RcCBS*	*tHMGR*, P*_*HXT*1_*- *ERG20*
CAS14	Strain CAS5 with integrated *PaGGPPS* and *RcCBS*	*tHMGR*, P*_*ERG*1_*- *ERG20*, P*_*ERG*1_*- *ERG9*
CAS15	Strain CAS10 with integrated *PaGGPPS* and *RcCBS*	*tHMGR*, P*_*HXT*1_*- *ERG20*, P*_*ERG*1_*- *ERG9*

The 805 bp promoter region of *ERG1* (Table S2) was amplified from genomic DNA of *S. cerevisiae* S288C after lysis in 30 μL 0.2% SDS at 95°C for 5 min and clarification at 14,000 g for 5 min. iProof™ High-Fidelity DNA Polymerase was used according to the manufacturer's protocol with primer pair F_ERG1p_*Spe*I/ R_ERG1p_*Sac*II. The PCR product was cut with *Spe*I/*Sac*II, and inserted into the integration vector pUG72 (Gueldener et al., 2002) close to the URA3 marker flanked by loxP sites. Marker and promoter were amplified with primers pair F_ERG9int/ R_ERG9int and transformed in yeast for homologous recombination at the ERG9 site (Table 3).

Plasmid pCAS4 for replacement of the promoter region of *ERG20* (Table S2) was kindly provided by Michael Eichenberger. The plasmid already contained up- and down-tags for homologous recombination at the ERG20 site, a URA3 marker and the promoter of *CYC1* for exchange with the promoter of *ERG20*. P_*CYC*1_ was released from the plasmid with *Bgl*II and *Hind*III and replaced by promoters of *ERG1* and *HXT1*. P_*ERG*1_ was amplified from plasmid pCAS3 with primers pair F_ERG1p_*Bgl*II and R_ERG1p_*Hind*III. P_*HXT*1_ was amplified from an expression vector found in our labs with primers pair F_HXT1p_*Bgl*II and R_HXT1p_*Hind*III (Table 3). The PCR products were cut with *Bgl*II/*Hind*III, and inserted into the integration vector pCAS3 to replace P_*CYC*1_. Strains with successful replacement of native promoters of *ERG20* and *ERG9* were verified by diagnostic PCR.

**Table 3:** Oligonucleotides used for plasmid construction (restriction sites are underlined).

**Name**	**Description**
F_GGPPS_*Hind*III	cgaAAGCTTATGTTGTCTACTGGTTTGTCTTT GTCTCC
R_GGPPS_*Sac*II	AAGCACTCCGCGGTTAGTG
F_ERG1p_*Spe*I	cgaACTAGTGTCGAATACTACTATGACCGC
R_ERG1p_*Sac*II	acgCCGCGGCATGACCCTTTTCTCGATATGTT
F_ERG9int	GGTTTTGGGTTTAGTGCCTAAACGAGCA GCGAGAACACGACCACGCAGCTGAA GCTTCGTACGC
R_ERG9int	CTTCATCTCGACCGGATGCAATGCCAATT GTAATAGCTTTCCCATACTCAC TATAGGGAGACCGGC
F_ERG1p_*Bgl*II	ctAGATCTGTCGAATACTACTATGACCGC
R_ERG1p_*Hind*III	ctgAAGCTTATGACCCTTTTCTCGATATGTT
F_HXT1p_*Bgl*II	tcAGATCTCAAGTGCTGATAGAAGAATACCAC
R_HXT1p_*Hind*III	gcgAAGCTTGATTTTACGTATATCAACTAGTTGACG

### Yeast growth

Yeast batch cultures for production of metabolites were performed with the System Duetz (EnzyScreen, Heemstede, Netherlands) in an ISF-1-W shaker (Kuhner, Birsfelden, Switzerland) at 30°C, 300 rpm, and 5 cm shaking diameter. Pre-cultures were prepared by inoculation of single colonies into 3 mL of selective liquid SC medium in triplicates. Pre-cultures were grown for 24 h at 30°C, 160 rpm. Optical density at 600 nm (OD_600_) of a 1:40 dilution was measured in an Ultrospec 10 table top spectrophotometer (GE Healthcare, Little Chalfont, United Kingdom). Main cultures were inoculated in 2.5 mL of selective SC medium in a 24-deepwell microplate to an OD_600_ of 0.1 and incubated from 24 up to 120 h as stated.

### Sample preparation

Cells from a 2 mL volume were harvested by centrifugation at 4,000 rpm for 5 min in 2 mL screw cap tubes. Supernatants were discarded and pellets were mixed with 500 μL of methanol. Pellets were shaken for 10 min at 60°C and 1,500 rpm in a Thermo-Shaker TS-100 (Axonlab, Reichenbach an der Fils, Germany). Cell debris was removed by centrifugation at 4,000 rpm for 5 min and liquid phases were transferred to glass vials. The methanol extracts were evaporated for 24 h at room temperature under the fume hood, resolubilized in 500 μL hexane and used for analysis.

### Detection of diterpenes

Casbene and alcohols derived from FPP and GGPP were detected using gas chromatography–mass spectrometry (GC–MS). Quantification was carried out using the Agilent Technologies 7890a GC system (Agilent Technologies, Santa Clara, USA) equipped with a 5975 Mass Selective Detector (MSD). Sample volumes of 1 μL were injected in splitless mode at 250°C with the following GC program: 80°C for 2 min, raise to 220°C at 25°C/min, raise to 240°C at 3°C/min, ramp at rate 80°C/min to 300°C and held for 3 min. Helium was used as carrier gas with a constant flow rate of 1.2 mL/min. The MSD was operated at 70 eV in scan mode, with a range between 35 and 500 *m/z*. Diterpenes were identified by retention time and comparison with a mass spectral database (NIST version 2.0, Gaithersburg, MD, USA) and/or comparison with authentic standards. β-Caryophyllene (10 mg/L) was added in each sample as internal standard and calibration curves were generated to quantify the target compounds. In the case of geranylgeranyol an authentic standard of the compound was used, whereas casbene quantification was approximated by a standard curve generated using an authentic standard of the diterpene cembrene.

## Results

### Establishing casbene production in *S. cerevisiae*

In order to assess casbene (CAS) production in yeast, the casbene synthase gene from *R. communis* (*RcCBS*) was cloned as a codon optimized version under control of the promoter of *TEF1* in a yeast expression plasmid (“pCAS1”). The N-terminal chloroplast transit peptide, identified with the ChloroP software used at default settings (Emanuelsson et al., 1999) and needed for plastidial localization in plants, was removed (Kirby et al., 2010). Plasmid pCAS1 was introduced into yeast strain H-MEV, engineered for production of isoprenoids. H-MEV contained a chromosomally integrated copy of a truncated 3-hydroxy-3-methylglutaryl coenzyme A reductase (*tHMGR*) enabling high mevalonate pathway activity (Donald et al., 1997). The resulting strain (CAS1), was analyzed for production of casbene. Small amounts of the compound (~ 0.8 mg/L) could be detected after 72 h of growth (data not shown).

To boost casbene production, we co-expressed a heterologous GGPP synthase from the plant-pathogenic fungus *Phomopsis amygdali*. Toyomasu et al. (2007) found that synthesis of the diterpene fusicoccadiene in *P. amygdali* is catalyzed by the unusual chimeric enzyme fusioccadiene synthase (PaFS). This multifunctional enzyme can synthetize fusicoccadiene starting from IPP and DMAPP, through (1) a prenyltransferase domain, synthetizing GGPP from the C5 isoprene units, and (2) a terpene cyclase domain, catalyzing the cyclization of GGPP into fusicoccadiene.

We reasoned that the expression of the prenyltransferase domain from PaFS in yeast, catalyzing all steps from the C5 to the C20 precursor unit, could enhance the efficiency of this conversion, as it requires only one enzyme for the entire biosynthesis of GGPP. A construct encoding for the codon optimized prenyltransferase domain (residues 390–719 of PaFS) (Chen et al., 2016) was synthesized and cloned under control of the promoter of *PGK1* into pCAS2 yeast expression vector. pCAS2 was introduced together with pCAS1 (*RcCBS*) into H-MEV, generating strain CAS2. *PaGGPPS*+*RcCBS* co-expression gave rise to a peak with the same retention time and mass-spectrum of the authentic casbene standard (Figure S1A), while, as previously discussed, only trace amounts of the compound accumulated when expressing solely *RcCBS* (Figure S1B). A titer of 32 mg/L of casbene was reached in strain CAS2 (*PaGGPPS*+*RcCBS*) after 72 h of batch culture. The relatively high titer of casbene strongly suggested that *PaGGPPS* could considerably increase the supply of the GGPP precursor necessary for casbene production by RcCBS. This suggestion was further supported by the appearance of a pronounced peak in the chromatogram corresponding to GGOH (Figure S1B), the prenyl alcohol accumulating in engineered yeast cells due to endogenous phosphatase activities acting on the excess amounts of GGPP (Faulkner et al., 1999; Tokuhiro et al., 2009).

### Casbene production in engineered strains with engineered mevalonate pathway

To further improve casbene titers, we focused on downregulation of competing metabolic pathways. PaGGPPSp uses IPP and DMAPP to catalyze the consecutive condensation steps needed for GGPP synthesis and is in competition with the native farnesyl diphosphate synthase, Erg20p for the IPP/DMAPP pool. Moreover, FPP, produced by Erg20p, enters the ergosterol biosynthetic pathway by action of the squalene synthase Erg9p. Thus, in order to redirect the flux to diterpene production, we attempted dynamic control of either *ERG20*, or of *ERG20* in combination with *ERG9*, by replacing their native promoters with the ergosterol sensitive promoter P_ERG1._ This approach has recently been shown to positively affect amorpha-4-11-diene production in yeast (Yuan and Ching, 2015). The promoter of *ERG1* senses intracellular ergosterol levels and adjusts transcription of downstream genes to enable production of optimal levels of this essential metabolite. We constructed three strains containing (1) *ERG20* under control of P_*ERG*1_, (2) *ERG9* under control of P_ERG1_ and (3) both *ERG20* and *ERG9* under control of P_*ERG*1_ (Table 2, CAS3-5). All engineered strains also carried an integrated copy of *tHMGR* and were transformed with pCAS1 (*RcCBS*) and pCAS2 (*PaGGPPS*) to evaluate the effect of dynamic control of expression of *ERG20* and/or *ERG9* on casbene production. The resulting strains were designated as CAS6 (P_*ERG*1_-*ERG20*), CAS7 (P_*ERG*1_-*ERG9*) and CAS8 (P_*ERG*1_-*ERG20* and P_*ERG*1_-*ERG9*) (Table 2). CAS2, containing the native promoters of *ERG20* and *ERG9*, was used as a reference strain. Strains were analyzed for casbene production in time course experiments up to 120 h cultivation time.

**Figure 3:**
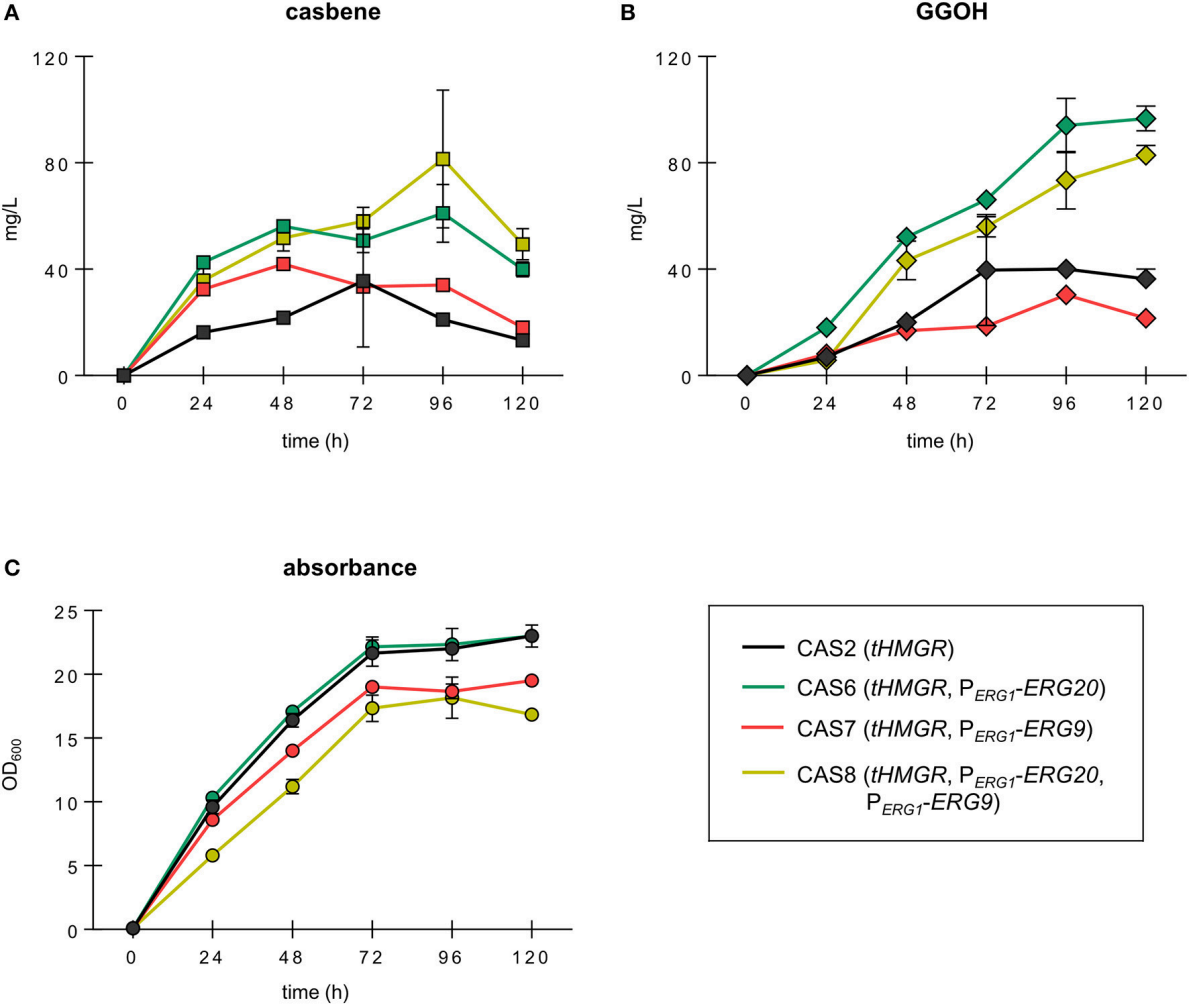
Growth and production of casbene and GGOH in engineered strains expressing *PaGGPPS* and *RcCBS*. Time course of production of casbene **(A)** and GGOH **(B)** in strains CAS2 (*tHMGR*), CAS6 (*tHMGR*, P_*ERG*1_-*ERG20*), CAS7 (*tHMGR*, P_*ERG*1_-*ERG9*), and CAS8 (*tHMGR*, P_*ERG*1_-*ERG20*, P_*ERG*1_-*ERG9*). All strains episomally expressed *PaGGPPS* and *RcCBS*. **(C)** Growth profile of the strains determined using optical density data recorded at 600 nm (OD_600_). All strains were grown in selective medium for maximally 120 h. Represented are the average and standard deviation of three independent experiments.

Interestingly, strains with engineered *ERG20* and *ERG9* expression improved the casbene titers substantially but showed different profiles of compound accumulation (Figure 3A). The two strains CAS6 (P_*ERG*1_-*ERG20*) and CAS8 (P_*ERG*1_-*ERG20*, P_*ERG*1_-*ERG9*) showed similar profiles of casbene accumulation over time. Strain CAS6 (P_*ERG*1_-*ERG20*) steadily built-up casbene during 96 h of cultivation where the measured casbene titer reached 61 mg/L. This represented a significant ~3-fold improvement in production over the reference strain CAS2, which accumulated only 21 mg/L of casbene. At 120 h the casbene titer in CAS6 dropped to ~40 mg/L, indicating that potential degradation mechanisms or modifying enzymes start to act on the casbene molecule. Strain CAS8 (P_*ERG*1_-*ERG20*, P_*ERG*1_-*ERG9*) harboring dynamic control of expression of both *ERG20* and *ERG9* could reach the highest titer of 81.4 mg/L casbene after 96 h of cultivation, a ~4-fold improvement over production in CAS2. Strain CAS7 (P_*ERG*1_-*ERG9*) revealed instead the highest production after 48 h of cultivation (42 mg/L). Casbene titers steadily dropped to 18 mg/L thereafter. Finally, reference strain CAS2 (*tHMGR*) showed a gradual accumulation of casbene up 35 mg/L upon 72 h of culture, followed by a decline to 13.3 mg/L at 120 h.

Also, GGOH considerably accumulated in the engineered strains (Figure 3B). Strains CAS7 (P_*ERG*1_-*ERG9*) and CAS2 (*tHMGR*) accumulated increasing amounts of GGOH during 72 h of cultivation, reaching up to 39.7 mg/L, and 30.3 mg/L, respectively. The titers of GGOH did not drop significantly throughout the entire cultivation period of 120 h. Strains CAS6 (P_*ERG*1_-*ERG20*) and CAS8 (P_*ERG*1_-*ERG20*, P_*ERG*1_-*ERG9*) showed a progressive build-up of the compound reaching, upon 120 h of growth, 96.7 mg/L and 82.9 mg/L, respectively.

Strain CAS6 (P_*ERG*1_-*ERG20*) in fact not only accumulated high amounts of both casbene and GGOH, but also showed optimal robustness, as revealed by its growth profile, which was comparable to that of reference strain CAS2 (Figure 3C). Strains with dynamic control of *ERG9* expression instead displayed growth retardation in comparison to reference strain CAS2. At the end of cultivation, strains CAS7 (P_*ERG*1_-*ERG9*) and CAS8 (P_*ERG*1_-*ERG20*, P_*ERG*1_-*ERG9*) reached cell densities which were lower compared to strain CAS2 by 15 and 27%, respectively.

These results demonstrated the potential of dynamically controlling FPP synthesis for improved casbene production via modification of the native *ERG20* promoter.

### Construction of stable *S. cerevisiae* strains for casbene production

To provide a stable chassis for casbene production, the engineered GGPP synthase *PaGGPPS* and the truncated casbene synthase *RcCBS* were stably integrated into the yeast chromosomes. In the previous plasmid-based assays, dynamic control of solely *ERG9* expression showed the poorest performance in terms of production and was for this reason not included in the next phase of experiments. As dynamic control of *ERG20* expression was shown to be crucial for improved diterpene production, we further investigated the effect of dynamic regulation of *ERG20* by replacing its native promoter with the glucose-sensing promoter of *HXT1* (P_*HXT*1_). It has recently been shown that using the HXT1 promoter to control *ERG9* expression could redirect the carbon flux from sterol synthesis toward sesquiterpene production (Scalcinati et al., 2012). The effect of expression of *ERG20* under control of P_*HXT*1_ was tested either alone, or in combination with control of expression of *ERG9* by P_*ERG*1_ (Table 2, strains CAS9 and CAS10).

The expression cassettes for *RcCBS* and *PaGGPPS* were integrated into five strains: CAS11 (*tHMGR*), CAS12 (*tHMGR*, P_*ERG*1_-*ERG20*), CAS13 (*tHMGR*, P_*HXT*1_-*ERG20*), CAS14 (*tHMGR*, P_*ERG*1_-*ERG20*, P_*ERG*1_-*ERG9*) and CAS15 (*tHMGR*, P_*HXT*1_-*ERG20*, P_*ERG*1_-*ERG9*). All strains were analyzed for casbene and GGOH accumulation upon growth in batch culture for 96 h, as this production time yielded the highest titers of casbene in plasmid-based assays.

**Figure 4:**
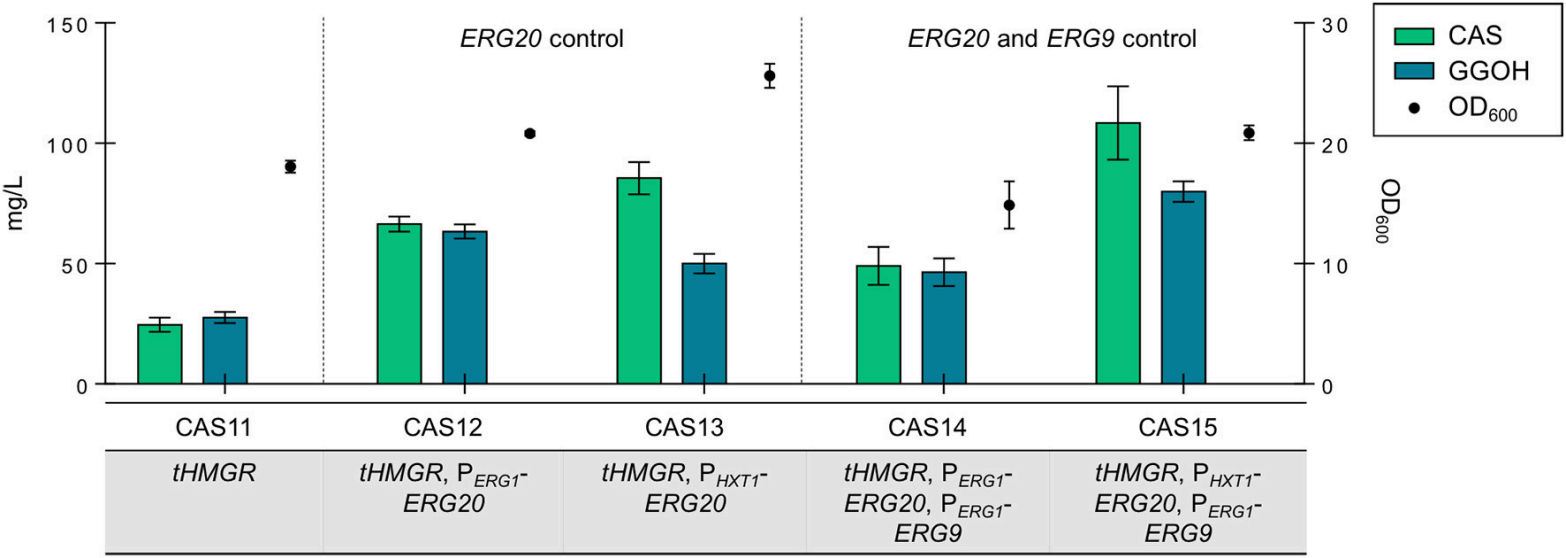
Production of casbene and GGOH in engineered strains containing integrated *PaGGPPS* and *RcCBS*. Casbene (green bars) and GGOH (blue bars) produced by strains CAS11 (*tHMGR*), CAS12 (*tHMGR*, P_*ERG*1_-*ERG20*), CAS13 (*tHMGR*, P_*HXT*1_-*ERG20*), CAS14 (*tHMGR*, P_*ERG*1_-*ERG20*, P_*ERG*1_-*ERG9*), and CAS15 (*tHMGR*, P_*HXT*1_-*ERG20*, P_*ERG*1_-*ERG9*). The corresponding OD_600_ values are represented by filled circles. Engineered strains were incubated in selective SC medium for 96 h (all data: mean ± SD, *n* = 3).

All engineered strains accumulated higher amounts of casbene and GGOH compared to reference strain CAS11 (24.6 mg/L of casbene and 27.6 mg/L of GGOH) (Figure 4). Strain CAS13 accumulated 85.5 mg/L of casbene and 50 mg/L of GGOH, whereas strain CAS12 accumulated 66.5 mg/L of casbene and 63.4 mg/L of GGOH, respectively. In strain CAS14 (*tHMGR*, P_*ERG*1_-*ERG20*, P_*ERG*1_-*ERG9*) accumulation of casbene did not reach the amounts observed previously (only 49.1 mg/L of casbene and 46.5 mg/L of GGOH accumulated). In strain CAS15 expression of *ERG20* controlled by P_*HXT*1_ combined with control of *ERG9* expression by promoter P_*ERG*1_ led to the highest titers of casbene (108.5 mg/L) and GGOH (79.9 mg/L) production. Interestingly, all strains, except CAS14, grew better than the reference strain CAS11 (*tHMGR* only). CAS14 (*tHMGR*, P_*ERG*1_-*ERG20*, P_*ERG*1_-*ERG9*) reached, in accordance with the plasmid-based expression results, lower final cell densities.

## Discussion

In this study we focused on the redirection of carbon flux toward casbene production by (1) introduction of a new biosynthetic branch for production of GGPP starting from IPP and DMAPP and (2) dynamic control of *ERG20* and *ERG9* expression by means of glucose- and ergosterol-sensitive promoters.

To increase the pool of GGPP in yeast, we expressed the GGPP synthase domain of the fusioccadiene synthase from *P. amygdali*. Similarly to other GGPP synthases from higher plants and bacteria (Vandermoten et al., 2009), this enzymatic domain synthesizes GGPP starting from one molecule of DMAPP and three molecules of IPP (Chen et al., 2016). In the yeast native pathway, GGPP is formed from the condensation of IPP and FPP, by action of the endogenous GGPP synthase Bts1p. FPP, in turn, is formed by the successive condensation of DMAPP with IPP units, catalyzed by FPP synthase, Erg20p. This requirement for two enzymatic steps for the production of GGPP, represents a limiting factor, partially due to the low native activity of Bts1p (Jiang et al., 1995). Low-titer diterpene production starting from the endogenous GGPP pool has been previously reported (Zhou et al., 2012; Ignea et al., 2015) and was confirmed in this study by the very low levels of casbene produced in yeast expressing *RcCBS* (Figure S1B). Improved efficiency of the conversion of FPP to GGPP was shown to significantly benefit diterpene production (Zhou et al., 2012; Ignea et al., 2015). Expression of *PaGGPPS* in yeast introduced the direct conversion of IPP and DMAPP to GGPP and improved the efficiency of GGPP formation. Indeed, expression of *PaGGPS*+*RcCBS* lead to 32 mg/L casbene accumulation, a ~40 fold improvement compared to cells expressing solely *RcCBS* and harnessing the endogenous source of GGPP. The substantial accumulation of GGOH in those cells clearly indicated the improved efficiency of GGPP biosynthesis and suggested that the flux toward diterpene synthesis could be further optimized.

To further increase the supply of IPP and DMAPP, we restricted the flux toward FPP and sterols, by limiting the activity of Erg20p and Erg9p. These two enzymes catalyze FPP synthesis and the first step in sterol biosynthesis, respectively. Sterols are essential for yeast growth, therefore it is crucial to ensure the delivery of intermediates and end-products at levels that can guarantee proper cell function. Ergosterol, the major product of sterol biosynthesis, fulfills in fact several vital cellular functions that require balanced sterol concentrations (Parks et al., 1995). Downregulation of Ergosterol synthesis by means of weak or inducible and repressible promoters has been shown to be beneficial for isoprenoid production, while negatively affecting yeast viability (Paradise et al., 2008; Fischer et al., 2011; Scalcinati et al., 2012; Ignea et al., 2014). Therefore, we engineered a dynamic control strategy to balance metabolism between diterpene formation and cell growth. P_*ERG*1_ represents an ergosterol-sensitive promoter, previously shown to efficiently restrict *ERG9* expression levels in order to boost amorphadiene production (FPP-derived) (Yuan and Ching, 2015). We applied a similar strategy to improve diterpene synthesis, extending dynamic control to *ERG20*, given the need to boost the IPP and DMAPP supply. Indeed, in our hands, the dynamic control of *ERG9* alone did not have the remarkable improvement in sesquiterpene production that was reported by Yuan and Ching (2015). The strains harboring such a modification (*tHMGR*, P_*ERG*1_-*ERG9*) produced amounts of casbene comparable to the reference strain (*tHMGR*), at least in plasmid-based assays. Moreover, they accumulated the lowest titer of GGOH and reached lower cell densities. Enhancement of the mevalonate pathway (by *tHMGR* overexpression), together with *ERG9* down-regulation in the absence of a sesquiterpene synthase or another draining route, possibly led to accumulation of toxic concentrations of pathway intermediates, such as FPP. The toxicity of isoprenoid precursors (including FPP) has previously been reported in *E. coli* (Martin et al., 2003; Sarria et al., 2014).

Dynamic control of *ERG20* by P_*ERG*1_ showed instead to be a significant improvement, leading to a 3-fold increase in production, without negatively affecting growth. Titers reached even 4-fold higher values than the control strain when both *ERG9* and *ERG20* expression were controlled by P_*ERG*1_. Downregulation of *ERG20* alone, similar to the *ERG9* downregulation reported by Yuan and Ching, most likely created a metabolic balance in which gene expression was adjusted to the cellular requirement for ergosterol biosynthesis. Once the cell sensed an excess of ergosterol, *ERG20* expression was decreased, resulting in redirection of the metabolic flux from IPP/DMAPP to GGPP and non-native diterpenoids. The concerted downregulation of both *ERG20* and *ERG9*, although leading to the highest casbene accumulation, might have resulted in an excessively restricted flux toward ergosterol which would explain the growth impairment observed upon dynamic control of both *ERG9* and *ERG20*, leading to a 27% reduced cell density.

Integration of the expression constructs for *PaGGPPS* and *RcCBS* in neutral loci of the yeast genome, together with dynamic control of *ERG20* by means of the glucose-sensitive promoter of *HXT1*, further enhanced production of casbene. P_*HXT*1_ allows for moderate expression levels when glucose is present in the medium, but leads to gene repression when glucose concentration is low or absent. This promoter was previously used for dynamic control of *ERG20* expression in strains engineered for improved production of geraniol (Zhao et al., 2017). When used for dynamic control of *ERG20*, P_*HXT*1_ had a beneficial effect on both production and growth compared to P_*ERG*1_. Repression of expression by P_*HXT*1_ might be stronger than the one imposed by P_*ERG*1_, leading to a higher supply of DMAPP and IPP for the heterologous GGPP synthase, thus explaining the higher titers. Moreover, since repression of expression mediated by P_*HXT*1_ occurs when glucose concentration is low, its control possibly maximized the carbon flux to ergosterol and biomass, thus explaining the higher cell densities. Combining dynamic control of *ERG20* by P_*HXT*1_ with dynamic control of *ERG9* by P_*ERG*1_ led to the highest casbene titer of 108.5 mg/L and worked best to guarantee a proper flux distribution between cell growth and diterpene synthesis.

Proper adjustment and usage of carbon source sensitive promoters, to improve casbene or in general diterpenoid production, opens up new perspectives for an extensive optimization of the culturing conditions during fermentation that were beyond the scope of this study. For example, substantial improvements in isoprenoid biosynthesis have been reported when ethanol is provided as the carbon source (Westfall et al., 2012; Zhao et al., 2017). In the future, it would therefore be interesting to analyze the effect of combining feeding with ethanol or with mixture of different carbon sources with *ERG20* expression under control of P_*HXT*1_ for casbene production.

Overall these results showed remarkable improvements in diterpene production via an engineered heterologous route toward synthesis of GGPP, combined with dynamic regulation of competing pathways. However, a substantial accumulation of GGOH could still be observed in all our engineered strains, indicating that not all of the GGPP produced is channeled toward casbene biosynthesis. This may be due to the low efficiency of the casbene synthase which, similarly to other enzymes of the secondary metabolism of plants, has not evolved to synthesize high titers of products. During preparation of this manuscript, a casbene production of 160 mg/L was reported in a yeast strain expressing multiple copies of casbene synthase constructs, with improved solubility achieved by protein tagging strategies (Wong et al., 2017). Titers in our strains could most likely similarly be improved by increasing the copy number of the casbene synthase gene. Moreover, fusion between the GGPP synthase *PaGGPPS* and the casbene synthase *RcCBS* could possibly offer an enzyme with improved solubility and a more efficient substrate channeling that mimics the natural chimera fusicoccadiene synthase. Expression of such a chimera may further increase production levels.

In conclusion, although further optimization is needed, the design developed here provides a valuable strategy for sustainable production of casbene derived chemicals. Moreover, the approach can easily be adapted to synthesis of other GGPP-derived compounds.

## Author contributions

RC designed experiments, performed strain constructions, extractions, analysis of samples, and drafted the manuscript. DR established the analysis methods. YM helped in construction of the integration strains. RC and HH coordinated the study. HH reviewed and edited the manuscript. All authors read and approved the final manuscript.

### Conflict of interest statement

All authors were or are employees of the company Evolva SA. Evolva SA is listed on the Swiss stock exchange.

## Abbreviations

MVA: mevalonate
IPP: isopentenyl diphosphate
DMAPP: dimethylallyl diphosphate
GPP: geranyl diphosphate
FPP: farnesyl diphosphate
GGPP: geranylgeranyl diphosphate
GGPPS: geranylgeranyl diphosphate synthase
*PaGGPPS*: geranylgeranyl diphosphate synthase from *Phomopsis amygdali*
*RcCBS*: casbene synthase from *Ricinus communis*
CAS: casbene
OD_600_: optical density at 600 nm
PaFS: fusicoccadiene synthase from *Phomopsis amygdali*
ORF: open reading frame.
