# Supplementary material for: Dynamic Control of ERG20 and ERG9 Expression for Improved Casbene Production in Saccharomyces cerevisiae
Source: Front Bioeng Biotechnol. 2018 Nov 1;6:160. doi: 10.3389/fbioe.2018.00160 (PMC6221901; doi:10.3389/fbioe.2018.00160)
Supplement: Supplementary file 1 [file Table_1.DOCX]

Table S1. Coding sequences of genes used in this study.

| Gene | Organism | Accession number | Sequence |
| --- | --- | --- | --- |
| *RcCBS* | *Ricinus communis* | XP_002513340 | atggaatacggtaacaacagattcccattcttctcttcatccgccaaatctcattttaagaaacctacccaagcctgcttgtcatctactactcatcaagaagttagaccattggcttactttccaccaactgtttggggtaatagattcgcttctttgactttcaacccatccgaatttgaatcctacgacgaaagagttatcgtcttgaagaagaaggttaaggacatcttgatctcttccacctctgattctgttgaaaccgttatcttgatcgacttgttgtgtagattgggtgtttcctaccatttcgaaaacgacatcgaagaattattgtccaagatcttcaactcccaaccagatttggttgacgaaaaagaatgtgacttgtacactgctgctatcgttttcagagtttttagacaacatggtttcaagatgagttccgacgttttctctaagttcaaggattccgacggtaaattcaaagaatcattgagaggtgatgccaagggtatgttgtctttgtttgaagctagtcacttgtccgttcatggtgaagatattttggaagaagccttcgctttcaccaaggattacttgcaatcttccgctgttgaattattcccaaacttgaagagacatatcaccaacgctttggaacaaccatttcattctggtgttccaagattggaagccagaaagttcattgacttgtatgaagccgatatcgaatgcagaaacgaaaccttgttggaatttgccaagttggattacaacagagtccaattattgcaccaacaagaattgtgtcaattctccaaatggtggaaggatttgaacttggcttctgatattccatacgccagagatagaatggccgaaattttcttttgggctgttgctatgtacttcgaaccagattatgctcacactagaatgattatcgccaaggttgttttgttgatttccttgatcgatgataccattgatgcttacgctactatggaagaaacccatattttggctgaagctgttgcaagatgggatatgtcttgtttggaaaagttgccagattacatgaaggtcatctacaagttgttgttgaacaccttctccgaattcgaaaaagaattgaccgctgaaggtaagagttactctgttaagtatggtagagaagcctttcaagaattggttagaggttactacttggaagctgtttggagagatgaaggtaaaattccatccttcgatgactacttgtacaacggttctatgactactggtttgcctttggtttctactgcttcttttatgggtgttcaagaaatcaccggtttgaacgaatttcaatggttggaaactaacccaaagttgtcttatgcttccggtgctttcattagattggttaacgatttgacctcccacgttactgaacaacaaagaggtcacgttgcatcttgtattgactgctacatgaatcaacacggtgtttctaaagatgaagccgttaagatcttgcaaaagatggctactgattgctggaaagaaatcaacgaagaatgcatgagacaatcccaagtttctgttggtcatttgatgagaattgtcaacttggctagattgaccgacgtttcttacaaatatggtgatggttacaccgattcccaacaattgaagcaatttgtcaagggtttgttcgttgacccaatctccatttga |
| *PaGGPPS* (390-719) | *Phomopsis amygdali* | A2PZA5.1 | atgttgtctactggtttgtctttgtctccagttcattctaatgaaggtaaggacttgcaaagagttgataccgatcatatcttcttcgaaaaggctgttttggaagctccatacgattacattgcttctatgccatctaagggtgtcagagatcaattcattgatgctttgaacgattggttgagagttccagatgttaaggttggtaagattaaggatgctgttagagtcttgcacaactccagtttgttgttggatgatttccaagacaactccccattgagaagaggtaaaccatctacccataacattttcggttctgctcaaactgttaacactgctacctactccattattaaggccattggtcaaatcatggaattctctgctggtgaatctgttcaagaagtcatgaactccatcatgatcttgtttcaaggtcaagccatggatttgttctggacttataatggtcatgttccatccgaagaagaatattacagaatgatcgaccaaaagaccggtcaattattctctattgctacctccttgttgttgaacgctgctgataacgaaattccaagaactaagatccaatcctgcttgcatagattgaccagattattaggtagatgcttccaaatcagagatgactaccaaaacttggtttctgctgattacactaagcaaaagggtttctgcgaagatttggacgaaggtaaatggtctttggctttgattcatatgatccacaagcaaagatcccatatggccttgttgaatgttttgtccactggtagaaaacatggtggtatgactttggaacaaaagcaattcgttttggacatcattgaagaagaaaagtccttggactacaccagatctgttatgatggacttgcatgttcaattgagagccgaaattggtagaatcgaaatcttgttggattctccaaatccagccatgagattgttgttagaattattgagagttcatcatcaccatcaccactaa |

Table S2. Sequences of promoters used in this study.

| Promoter | Sequence |
| --- | --- |
| P*_ERG1_* | tgtcgaatactactatgaccgctttttagaatcgtacgacaacggtgaccacttgattggtctgggggtcctacaacttgattttatcgttgaaaacaagaatatagacagccttcttgccaactcttatttgcaccagcaaagaggcggtgcaatcatcagtaatacaggacttgtctcgcaagatacgaccaagccgtactacgttcgggatttaatcttctcgcagtctgcaggcgccttgagatttgcgttcggcctaaacgtttgctccacaaacgtgaatggtatgaacatggacatgagcgtggttcagggcactctacgggatcgtggcgaatgggaatcgttctgcaagctcttctaccaaaccatcggcgaatttgcgtcgctttaatgcgatactgccgtagcgggccttcgtatagctcggccgagctcgtacaaaaggcaagcagtgtatcggacagagctgatataacacaatacgctcgtagtcgatgcatgccgtggctgctctcggtcgggtataagtcttagacaatagtcttacctcgcatgtataataaatcttttgtatttaatctattatatgtttctatgcttttttttcctattgttgtttgcttttccttttccttatttctttctagcttctaattttctttcttttttttttttttcattgaaaattatatatatatatatatatcagaacaattgtccagtattgaacaatacaggttatttcgaacaattgaaaaaaaaaaatcacagaaaaacatatcgagaaaagggtcataagct |
| P*_CYC1_* | gcgttggttggtggatcaagcccacgcgtaggcaatcctcgagcagatccgccaggcgtgtatatatagcgtggatggccaggcaactttagtgctgacacatacaggcatatatatatgtgtgcgacaacacatgatcatatggcatgcatgtgctctgtatgtatataaaactcttgttttcttcttttctctaaatattctttccttatacattaggacctttgcagcataaattactatacttctatagacacacaaacacaaatacacacactaaattaata |
| P*_HXT1_* | tcaagtgctgatagaagaataccactcatatgacgtgggcagaagacagcaaacgtaaacatgagctgctgcgacatttgatggcttttatccgacaagccaggaaactccaccattatctaatgtagcaaaatatttcttaacacccgaagttgcgtgtccccctcacgtttttaatcatttgaattagtatattgaaattatatataaaggcaacaatgtccccataatcaattccatctggggtctcatgttctttccccaccttaaaatctataaagatatcataatcgtcaactagttgatatacgtaaaatcaagct |
