# Supplementary figures and images for: Dynamic Control of ERG20 and ERG9 Expression for Improved Casbene Production in Saccharomyces cerevisiae
Source: Front Bioeng Biotechnol. 2018 Nov 1;6:160. doi: 10.3389/fbioe.2018.00160 (PMC6221901; doi:10.3389/fbioe.2018.00160)

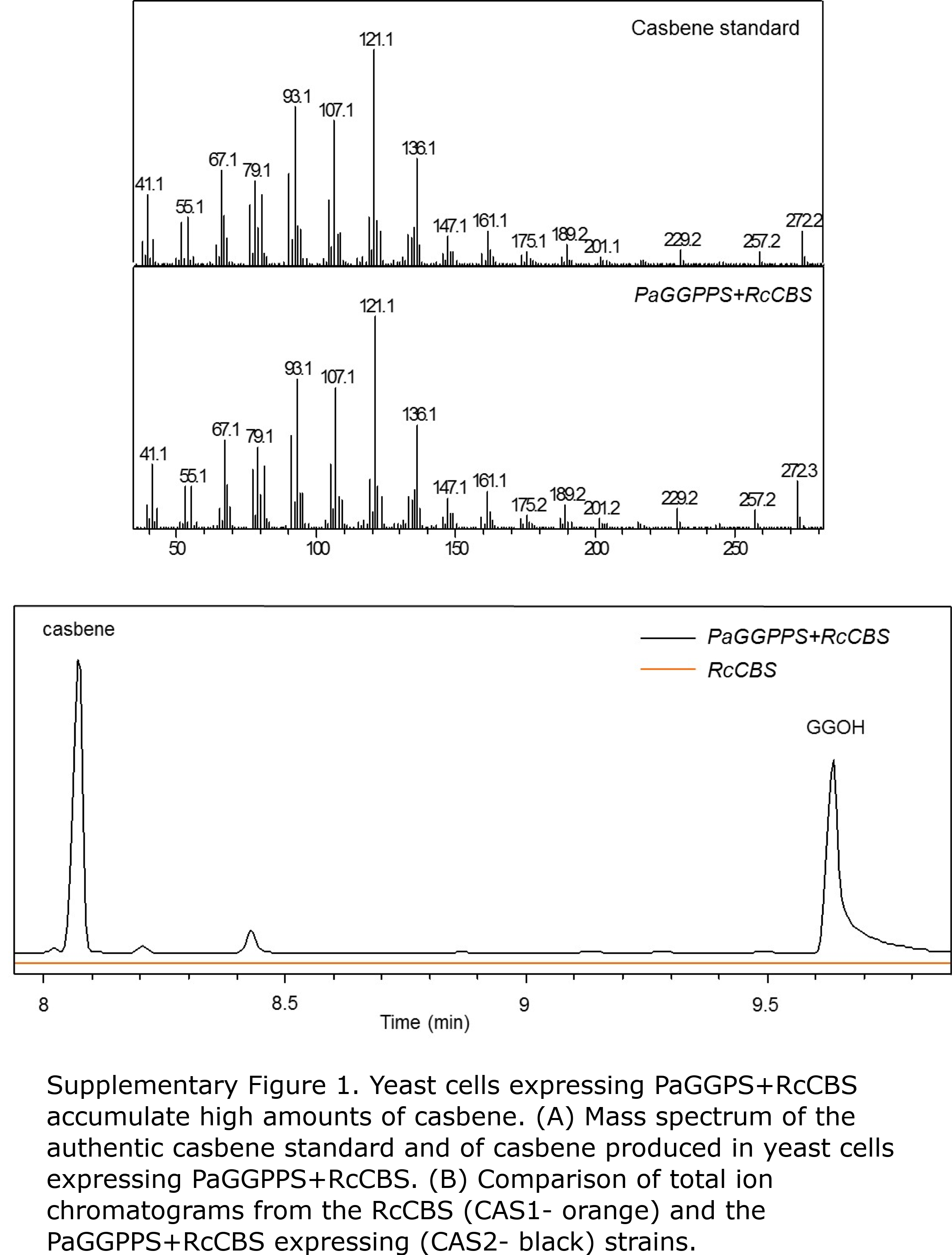

Supplement: Supplementary file 2 [file Image_1.TIFF]
